# Staging of post-settlement growth in the nudibranch *Hypselodoris festiva*

**DOI:** 10.1038/s41598-024-66322-4

**Published:** 2024-07-21

**Authors:** Makiko Hayashi, Hiroaki Nakano

**Affiliations:** Shimoda Marine Research Center, University of Tsukuba, 5-10-1 Shimoda, Shizuoka, Japan

**Keywords:** *Hypselodoris festiva*, Chromodorid, Nudibranch, Post-settlement, Rearing, Organogenesis, Doridina, Body coloration, Staging, Ontogeny, Zoology, Taxonomy

## Abstract

Sea slugs of the family Chromodorididae (Nudibranchia, Gastropoda, Mollusca) have garnered attention by researchers and hobbyists alike for their bright and variable color patterns. However, the chromodorid life cycle has yet to be fully elucidated as there exist no reports of their rearing in the laboratory. Here, we report the rearing of *Hypselodoris festiva* from eggs to adults, where we categorized their post-settlement growth patterns from juvenile to adult stages. Body coloration appeared around 36 days, and organogenesis of vital adult organs began within 42 days after hatching. The anus of *H. festiva* was observed to change from a ventral to dorsal position during juvenile growth. Individuals reached sexual maturity after six months post-hatching, with successful mating and spawning observed ex situ. This study outlines comprehensive rearing methods and life cycle staging that could be applied to other chromodorid species. We propose *H. festiva* as a model organism for chromodorid research, with this research contributing to the progress of developmental and evolutionary research on sea slugs.

## Introduction

Sea slugs are marine gastropods (phylum Mollusca) with differing degrees of shell degeneration, with their shells being either reduced, internalized, or completely lost^1^. They were traditionally clustered as “opisthobranchs”, but the monophyly of the clade is not supported by both morphological and molecular phylogenetic analyses^2–5^. It is now widely accepted that most, if not all, sea slugs are included in the subclass Heterobranchia, along with terrestrial snails within the class Gastropoda^1–3,6^. Sea slugs include diverse groups such as Nudibranchia, Aplysiida, Cephalaspidea, Sacoglossa, and Pleurobranchida^6^. All species within the order Nudibranchia show a complete loss of their shell and possess naked gills^7^. High morphological diversity is present in this order^8^, which is comprised of two suborders: Cladobranchia and Doridina^9^.

The family Chromodorididae (suborder Doridina) contains more than 395 species^10^, with many species possessing bright and variable color patterns thought to function as aposematic visual signals^11,12^. Hence, they have garnered fascination and attention by researchers and hobbyists alike^13,14^. Like all other nudibranchs, chromodorids possess rhinophores, which are a pair of chemosensory organs present near the anterior end of the body^1,15^. Near the posterior end are gills, composed of unipennate gill plumes surrounding the anal papillae^15^. At the mantle edge are mantle dermal formations (MDFs), sometimes called “mantle glands”, composed of large cells filled with a single vacuole which provide chemical defense against predators^16^. Although they lack shells, internal spicules have been reported in some species^17,18^. The importance of gill morphology and the arrangement of MDFs for species identification were emphasized by Rudman (1984)^19^. The morphological characteristics listed above together with radula shape were primarily used for chromodorid species identification. However, recent molecular phylogenetic analyses have led to major taxonomical revisions within Chromodorididae^20,21^.

Nudibranchs are simultaneous hermaphrodites, and mature individuals spawn fertilized eggs as egg masses after mating^1^. Most species undergo spiral cleavage, pass through the gastrula, develop into trochophore or trochophore-like larvae, and hatch as planktotrophic veliger larvae that last for several weeks^22,23^. The larval stage has been shortened in some species, with either extremely short free-swimming periods^24,25^ or with juveniles hatching from eggs^26^. Regarding nudibranch development with prolonged planktotrophic larval stages, there are only limited reports on the subsequent stages of settlement, metamorphosis, and juvenile growth^27–32^. Among the over 1,500 species of the suborder Doridina^10^, only one species with a planktotrophic larval stage, *Corambe obscura*, has been reared from eggs to adults in the laboratory^31^. Detailed observations of settlement and metamorphosis have been reported in dorid species with a free-living larval stage including *Adalaria proxima*^24^*, Rostanga pulchra*^28^*,* and *Onchidoris bilamellata*^22,33^. Concerning the family Chromodorididae, development prior to hatching has been reported in some species such as *Chromodoris quadricolor*^34^*, Doriprismatica atromarginata*^34^ and *Doriprismatica sibogae*^35^. Settlement and metamorphosis of species with prolonged planktotrophic larval stages^36^, and hatched juveniles with abbreviated larval stages^17,35,37^have been reported in this family. Yet, there are no reports of the growth process from juveniles to adults in the laboratory for any chromodorid species. This has been attributed to difficulties in identifying and obtaining the food for these chromodorids^38^, which is often necessary to induce settlement and metamorphosis in larvae^36^. Chromodorids have highly selective diets and species-specific prey^39^, and the adult food has not been identified in many chromodorid species. Furthermore, it is not easy to obtain metamorphosis-competent larvae for observing later developmental stages. Nudibranch planktotrophic larvae, including those of chromodorids, are reported to have high mortality rates in laboratory conditions, with the main causes being unknown diet and larval adherence to the water surface^22^. Hence, finding the suitable conditions for successful larval culture and establishing the conditions for settlement and metamorphosis are large obstacles for studies on chromodorid development.

This study aims to fill the ontogenetic knowledge gap in the chromodorid life cycle using the species *Hypselodoris festiva* (A. Adams, 1861)^40^, which is found widespread on the east and west coasts of Japan^41^. *H. festiva* together with its primary food, the sponge *Dysidea* sp., were collected from both the intertidal and subtidal zones of Shimoda, Japan. We outlined their life cycle and categorized their post-settlement growth into nine stages based on their organ and body color formation; both of which are easily identifiable external characteristics. We provide a comprehensive methodology for their rearing, for which we hope will form the basis for future studies on chromodorids.

## Results

### Reproduction and spawning

Adult *Hypselodoris festiva* individuals were observed to spawn one to two days following collection from the field. Mating began with one individual touching the other with their oral tentacles and mouth. These two individuals then made contact with the right sides of their bodies, where their reproductive organs are located (Supplementary Fig. S1A). During egg laying, the reproductive organs were thrusted out while the individual proceeded to slowly move in a counterclockwise direction (Supplementary Fig. S1B). The egg mass was spiral-shaped, with the first laid eggs contained within the inner area of the mass (Supplementary Fig. S1C). Agreeing with the previous descriptions by Baba et al.^42^, we observed these egg masses to be ribbon-shaped. One side of the ribbon attached to substratum while the other side had a wavy pattern (Supplementary Fig. S1D). The rows of egg capsules within the mass were folded together (Supplementary Fig. S1D, E) and were completely covered with mucus. Each egg capsule was observed to contain a single embryo (Supplementary Fig. S1F).

### Embryonic development

Embryonic development agreed with a previous report of development prior to hatching in *H. festiva*^43^ (Supplementary Fig. S2). The timing of cleavage differed between eggs within the same mass, yet gastrulation occurred synchronously two days after being laid. After about three days, cilia became prominent and the embryo became trochophore-like. After about four days, velum was formed and an early veliger stage was reached. After about five days, shell and operculum became visible in the veliger larvae. After around six days, slight movements were observed on the egg membrane near the velar cilia. Following this observation, the veliger hatched within a day. When egg masses were left intact, embryos at the outer layers were able to develop normally, but the inner layers exhibited slowed to halted development. When egg masses were chopped into pieces (see Supplementary Fig. S5C), hatching was observed from whole egg mass uniformly six days after the egg mass was laid. Incubation was successful at 22 °C, while time to hatching took twice as long at 20 °C (12 days), and no hatching occurred at 18 °C.

### Larval stage

Newly hatched veliger larvae had a shell and two-lobed velum and began to swim actively while feeding on the algae *Chaetoceros* sp. (Supplementary Fig. S3A, Supplementary Video 1). Shell length and days post-hatching showed a linear relationship (R^2^ = 0.9401), with shell length growth plateauing at around 20 days post-hatching (Supplementary Fig. S3B). Eye spots appeared in most individuals around this time. Two to three days after the formation of eye spots, the majority of larvae formed propodia and feet, becoming pediveligers (Supplementary Fig. S3C). After another two to three days, individuals were observed to crawl and gain the competence for metamorphosis. Time to eye spot formation varied across larval densities, while the timing of subsequent important developmental steps prior to the acquisition of metamorphosis competence remained consistent when incubated at 22°C.

### Developmental stages of *H. festiva* after settlement

We divided the post settlement growth process of *H. festiva* into nine stages: the metamorphosis phases M1 and M2 and the juvenile phases J1 to J7 (Table 1).

**Table 1:** *H. festiva* post settlement stages.

Phase	Stage	Days after hatching	Main characteristics	Other characteristics
Metamorphosis	M1	21	Settlement	Gliding by foot, degeneration of velum, casting of operculum, detorsion begins
	M2	21	Loss of shell	Detorsion continues, rhinophores formation begins
Juvenile	J1	25	Formation of spicules	Formation of posteroventral juvenile anus
	J2	30	MDFs appear at posterior mantle edge	Rhinophores become pigmented
	J3	36	Mantle pigmentation appears	Temporary thickening of the body
	J4	42	Formation of adult anus and anal papilla at posterodorsal side	Gill with three plumes
	J5	53	MDFs appear at anterior mantle edge	Gill plumes arranged in an arc
	J6	79	Rhinophores become spindle shaped	Blue spots appear on notum, gill plumes increase up to nine and arranged circularly
	J7	94	Appearance of yellow spots	11th gill plume appears
Adult		164	Gills have at least 11 plumes with the same length	Gill plumes increase to 12 or more, mating and spawning possible

#### Metamorphosis phase—Transition from planktonic to benthic life, with massive morphological changes including detorsion.

##### Stage M1—21 days post hatching: settlement

**Figure 1:**
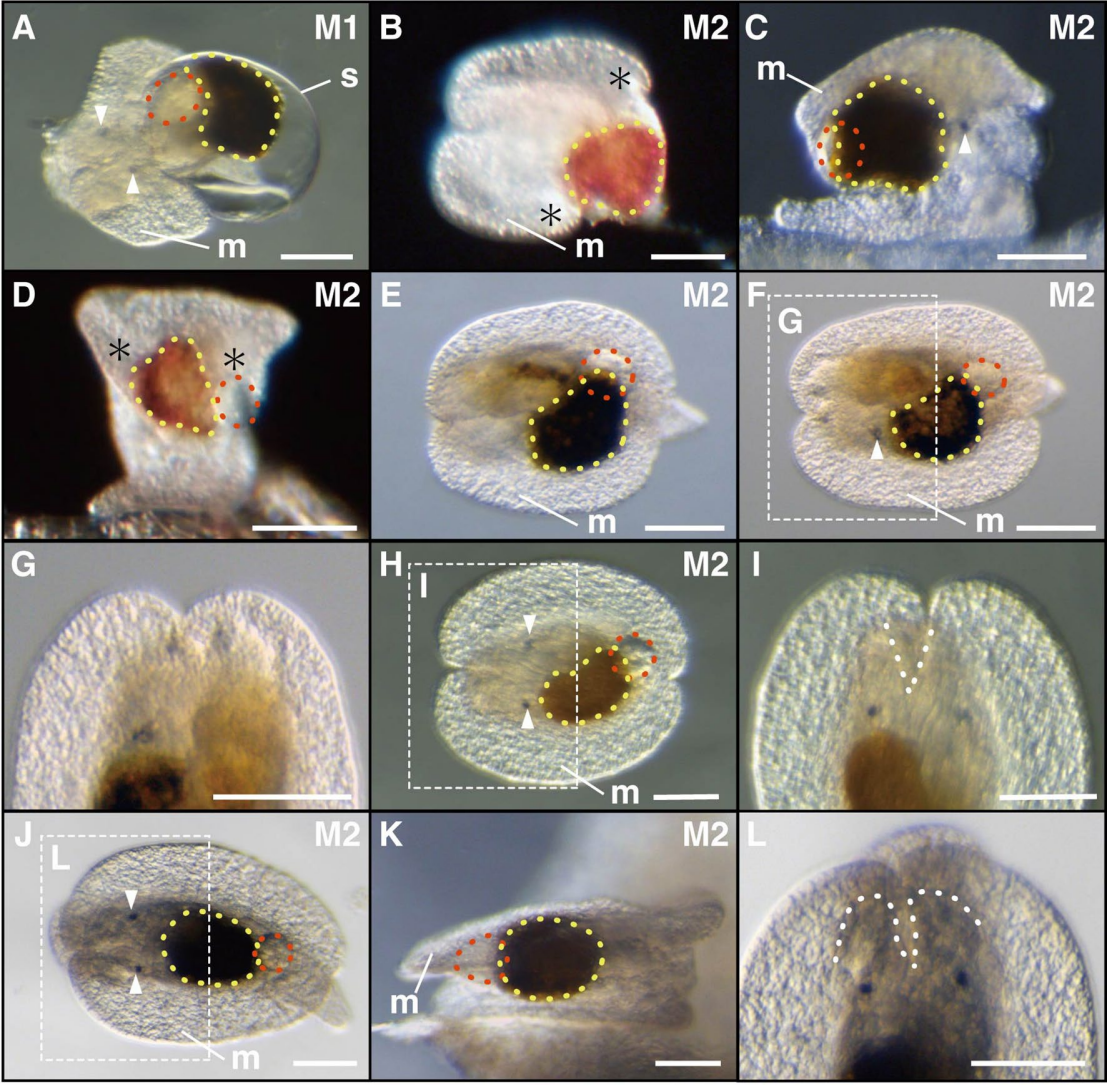
Metamorphosis of *H. festiva*. (**A**) Larva during shell casting. arrowheads; eye spots, yellow dotted line; digestive gland, red dotted line; anal complex, m; mantle, s; shell. (**B**–**D**) M2 stage individuals, just after shell casting. (**B**) Eye spots are not visible from the dorsal side. The posterior tip of the mantle is divided into two lateral lobes (*). Yellow dotted line; digestive gland. (**C**) Eye spot (arrowhead) is visible from the lateral side. (**D**) The anal complex (red dotted line) is to the right of the digestive gland (yellow dotted line). (**E**) Post-larva about ten minutes after shell casting with the mantle covering the entire visceral mass. Anal complex (red dotted line) is to the dorsal and right of the digestive gland (yellow dotted line). (**F**) Post-larvae three hours after settlement. Only the left eye spot (arrowhead) is visible dorsally. (**G**) Notch is not present at the anterior part of the mantle. (**H**) Post-larvae four hours after settlement. Both eyespots (arrowheads) are visible dorsally. Anal complex (red dotted line) is more posterior than in F. (**I**) A notch (dotted line) appears. (**J**, **K**) Post-larva one day after settlement. (**J**) The anal complex (red dotted line) is posterior to the digestive gland, along the midline of the body. (**K**) The body is flat compared to C. (**L**) Rhinophore rudiments (dotted line) appear on both sides of the notch, slightly anterior to the eye spots. (**A**, **B**, **E**, **F**, **H**, **J**) dorsal view, anterior to the left. (**G**, **I**, **L**) dorsal view, anterior to top. (**C**, **K**) right lateral view, anterior to the right. (**D**) posterior view. Scale bars: (100 μm).

Settlement marked the beginning of metamorphosis stage 1 (M1) (Fig. 1A, Supplementary Video 2). Individuals were observed crawling on the bottom of dishes or sponge pieces. The velum contracted into their shell and degenerated. Velar cilia detached and scattered, with the larval visceral mass separated from the shell soon after. This was followed by the casting off of the operculum and the shell.

##### Stage M2—21 days post hatching: loss of the shell

**Figure 2:**
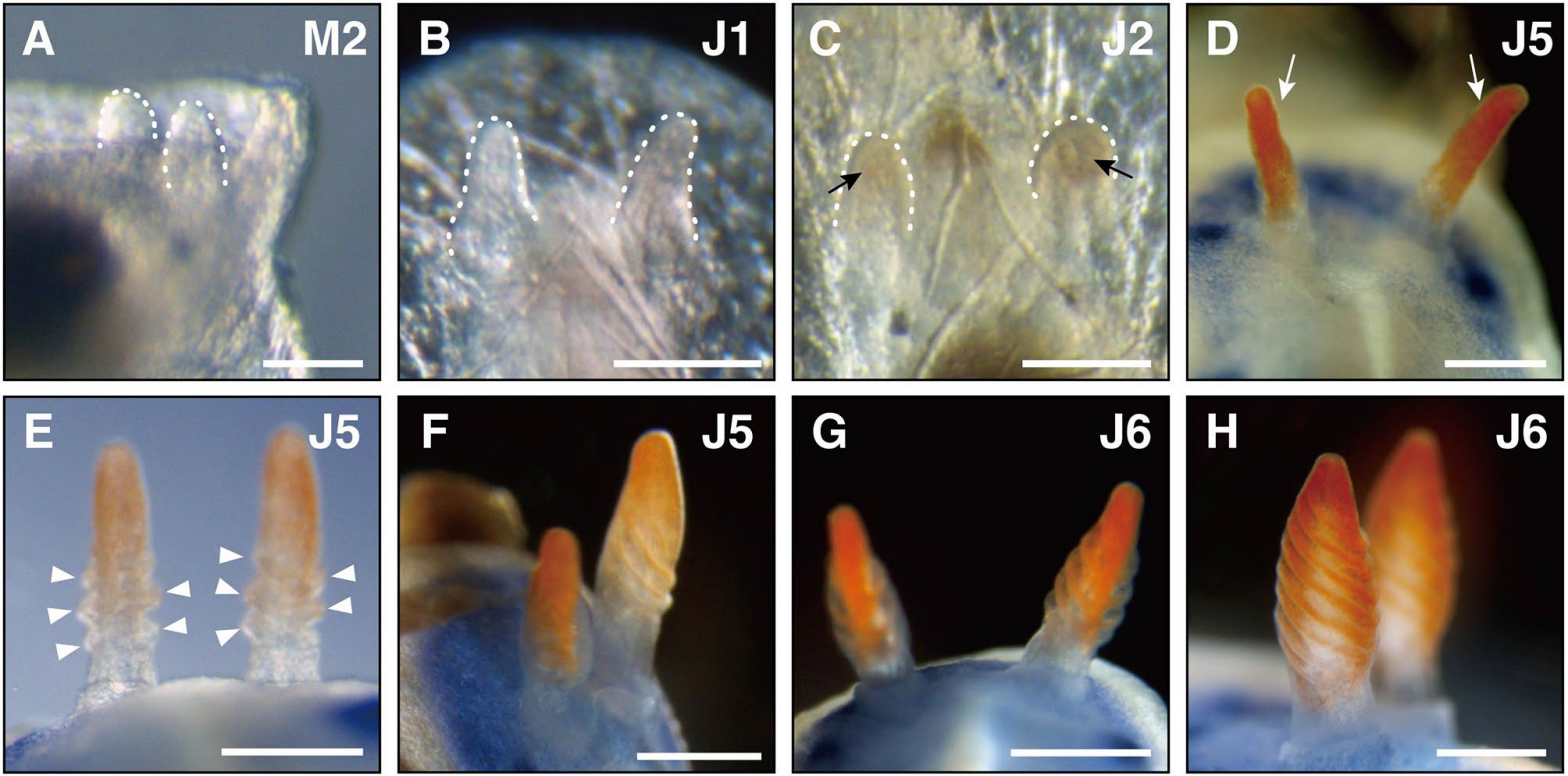
Formation of rhinophores in *H. festiva.* (**A**) Rhinophores (white dotted lines) appear. (**B**) Rhinophores have become longer (white dotted lines). (**C**) Vermilion pigmentation (black arrows) appear on the rhinophores (white dotted lines). (**D**) Rhinophores (white arrows) further elongated, with pigmentation present except for the basal part. (**E**) Ridges (white arrowheads) appear on the sides. (**F**) Ridges become more evident. (**G**) Rhinophores become spindle shaped with ridges forming on the surface. (**H**) Lamellate ridges and distinct stalks are present on the rhinophores. Scale bars: (**A**) (50 μm), (**B**) and (**C**) (100 μm), (**D**) and (**E**) (300 μm), (**F**–**H**) (500 μm).

Once the shell was cast off, individuals entered metamorphosis stage two (M2). Eye spots were unobservable from the dorsal side (Fig. 1B), but visible from the lateral sides (Fig. 1C). The mantle fold covered the posterior part of the body but not the entire visceral mass, with its posterior end divided into the right and left lobes (Fig. 1B, D). Parts of the visceral mass that remain exposed contained the digestive gland (Fig. 1B, D), derived from the left larval digestive gland. The anal complex containing the cells that form the future juvenile anal gland was to the right of the digestive gland (Fig. 1D). Ten minutes following the casting off of the shell, the posterior ends of the right and left mantle lobes extended posteriorly and covered the entire visceral mass (Fig. 1E). The anal complex on the right side of the body moved slightly to the posterior, and partially overlapped with the digestive gland (situated ventrally). From three hours following the casting off of the shell, eye spots became visible from the dorsal side (Fig. 1F-I). The anal complex remained on the right side of the body midline, and moved towards the posterior (Fig. 1F, H). At the anterior part of the mantle, a notch was observed to form (Fig. 1H, I). One day following the casting off of the shell, the digestive gland, originally on the left side of the body midline, moved towards the middle (Fig. 1J). The body was observed to flatten (compare Fig. 1 C and K) similar to other dorids during metamorphosis^24^, while the anal complex became smaller (compare Fig. 1H and J). The anal complex and digestive gland then contacted at the midline. Finally, rhinophores appeared on both sides of the anterior notch (Figs. 1L, and 2A).

#### Early juvenile phase: *H*. *festiva* begins to feed on sponges, the prey of adults. Adult organs begin to develop, and the juvenile ventral anus is present.

##### Stage J1—25 days post hatching: formation of spicules

**Figure 3:**
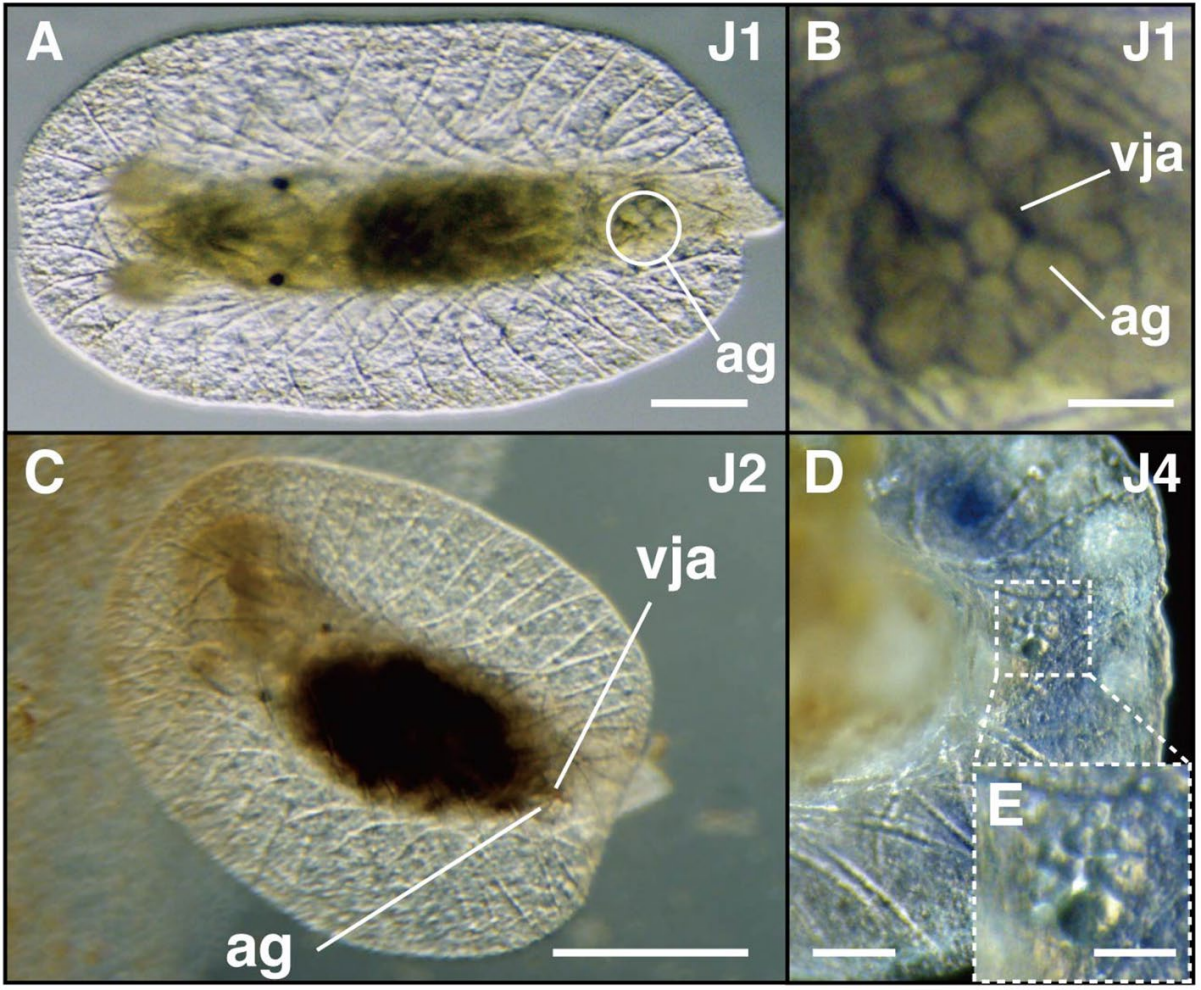
Ventral juvenile anus of *H. festiva*. (**A**) J1 stage juvenile. (**B**) Anal gland surrounding the ventral juvenile anus in a J1 juvenile. (**C**) J2 stage juvenile with the posterior end of the mantle lifted up during excretion from the ventral juvenile anus. (**D**) and (**E**) J4 stage juvenile with anal glands remaining posteroventrally. (**A**–**C**) dorsal view, anterior to the left. (**D**) ventral view, anterior to the left. Abbreviations: ag (anal gland), vja (ventral juvenile anus). Scale bars: (**A**, **D**) (100 μm), (**B**) (25 μm), (**C**) (300 μm), (**E**) (50 μm).

**Figure 4:**
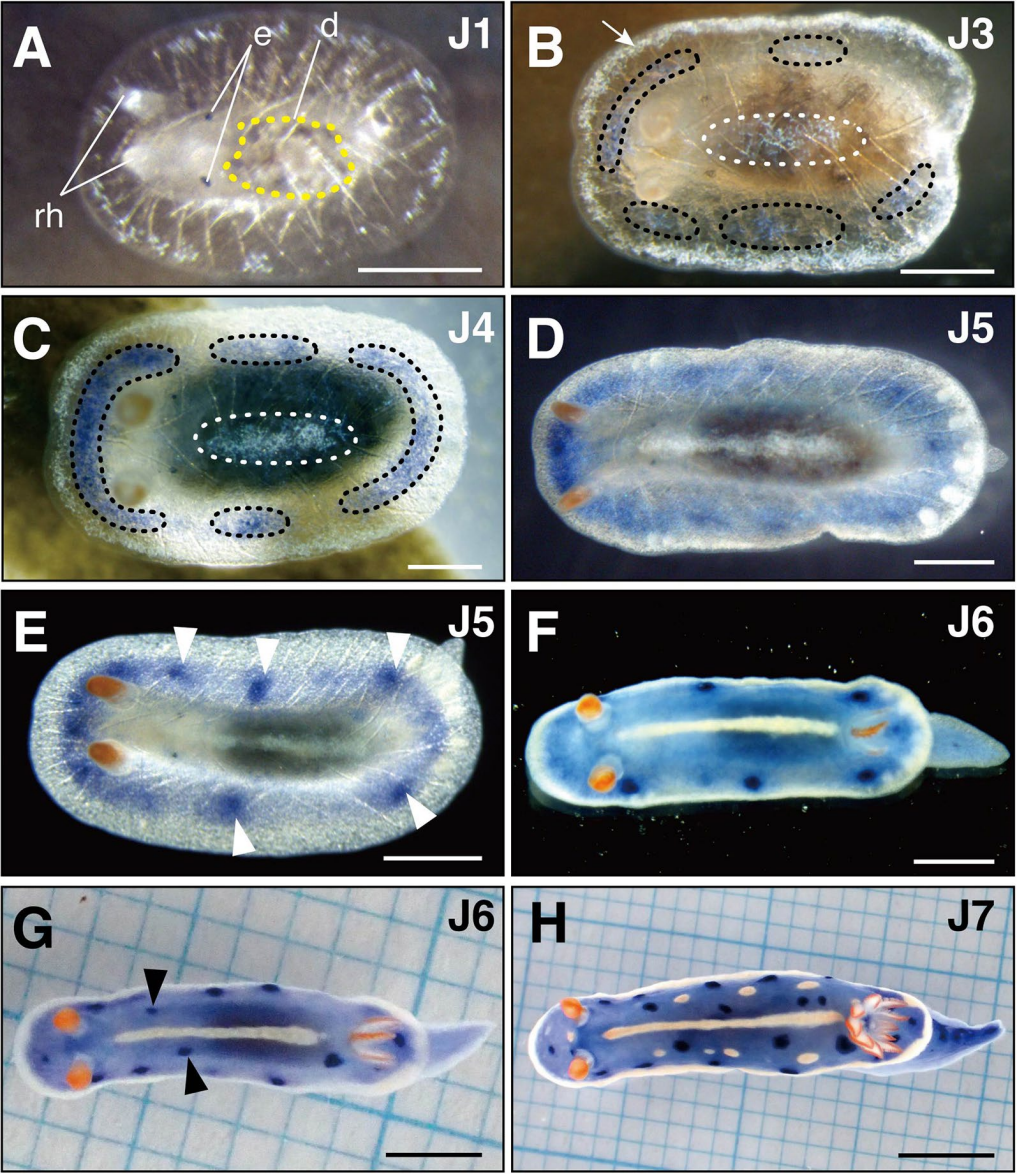
Body color formation in *H. festiva*. (**A**) Mantle is transparent. e (eye spots), d (digestive gland), rh (rhinophores) (**B**) Yellow pigmentation appears at the midline of the mantle (inside white dotted lines) and the mantle margin (arrow). Blue pigmentation seen on the dorsal side, interior to the yellow margin (inside black dotted lines). The yellow pigmentation is pictured whitish due to the strong light source of the microscope. (**C**) Pigmentation increases, and the coloration becomes darker. (**D**) Blue pigmentation spreads in a gradient toward the center of the notum with the center being lighter colored. (**E**) In some laboratory reared individuals, blue spots (white arrowheads) appeared slightly inside the yellow area at the periphery. (**F**) Blue coloration is present throughout the mantle and on the foot. (**G**) Blue spots further appear on the dorsal notum (black arrowheads). (**H**) Yellow spots appear between the mantle margin and the dorsal midline. (**A**–**H**), dorsal view, anterior to the left. Scale bars: (**A**–**C**) (200 μm), (**D**) and (**E**) (500 μm), (**F**) (1 mm), (**G**) (2 mm), (**H**) (4 mm).

The formation of spicules within the mantle marked the beginning of juvenile stage 1 (J1). Spicules formed a mesh-like structure when observed dorsally (Fig. 3A, Supplementary Fig. S4A, B), and were organized within three different layers: dorsal, middle, and ventral. Rhinophores elongated, but pigmentation was yet to be observed on the rhinophores during this stage (Fig. 2B). Individuals were able to retract the rhinophores freely within their rhinophore cavity (Supplementary Video 3). Within the posteroventral part of the mantle, the anal gland, composed of polygonal cells derived from the anal complex, was present (Fig. 3A, B, Supplementary Fig. S4B). At the center of the anal gland was the juvenile anus, which opened externally at the ventral side of the mantle (Fig. 3B). The mantle was transparent at this stage, while eye spots and digestive glands were visible (Fig. 4A).

##### Stage J2—30 days post hatching: MDFs appear at posterior mantle edge

**Figure 5:**
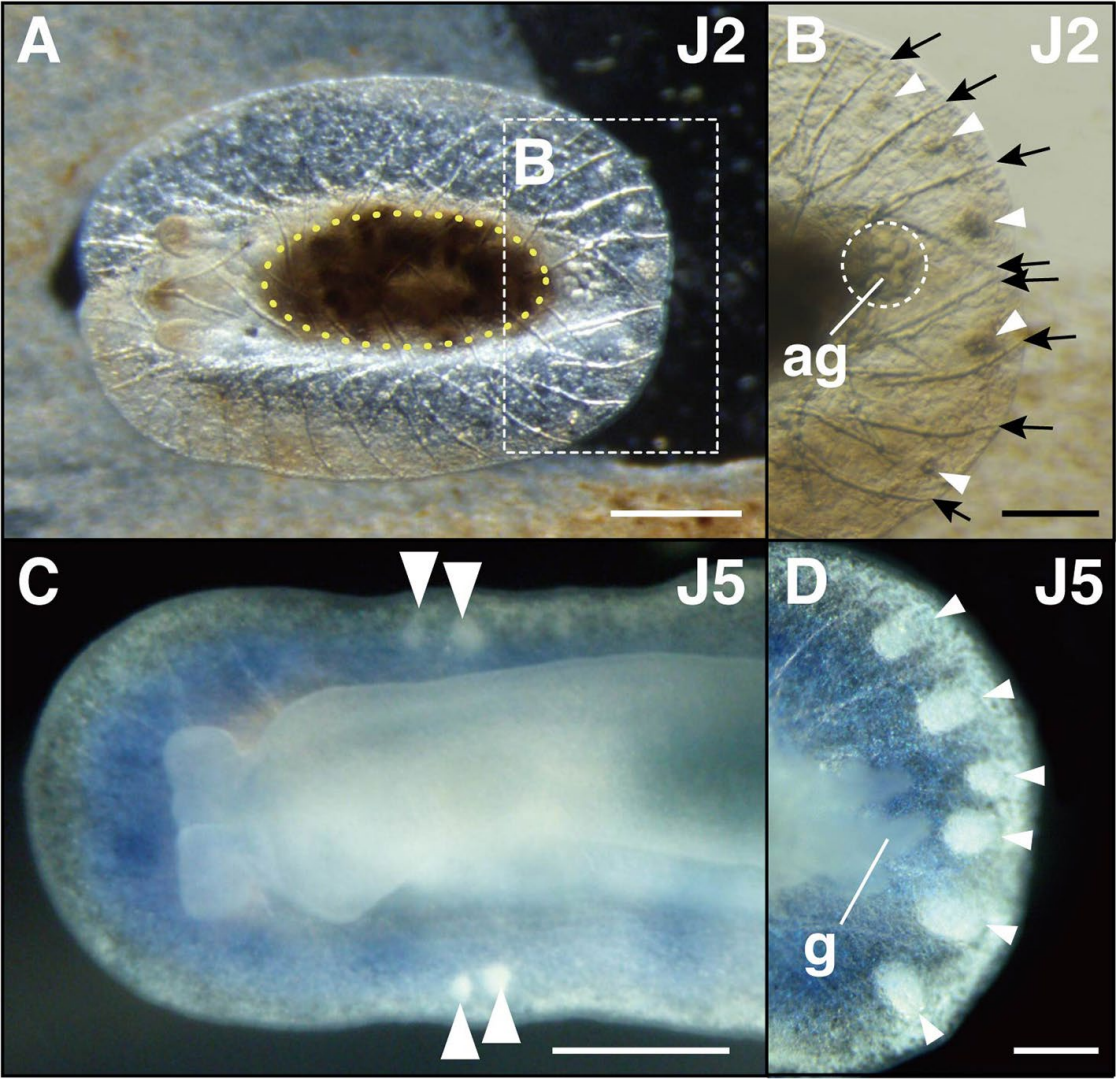
Formation of mantle dermal formations (MDFs) in *H. festiva*. (**A**, **B**) J2 stage juvenile. MDFs are present at the posterior mantle edge (dotted square). Digestive gland (yellow dotted line). (**B**) Multiple MDFs (arrowheads) are present between the spicules (arrows). (**C**) J5 stage juvenile, ventral view. MDFs (arrowheads) appear at the anterior lateral edges. (**D**) J5 stage juvenile. Large spherical MDFs (arrowheads) are present at the posterior mantle edge. All images: anterior to left, (**A**, **B**, **D**) dorsal view, (**C**) ventral view. Abbreviations: ag (anal gland), g (gill). scale bars: (**A**) (200 μm), (**B**) and (**D**) (100 μm), (**C**) (500 μm).

The formation of mantle dermal formations (MDFs) between the spicules at the posterior part of the mantle edge marked the beginning of juvenile stage two (J2) (Fig. 5A, B). Most of the body cavity was occupied by the digestive gland, which was often filled with food (Fig. 5A). The posterior part of the mantle occasionally became rolled up during excretion (Fig. 3C, Supplementary Video 4). Slight vermilion pigmentation was present on the rhinophores (Fig. 2C). Juveniles at this stage were largely immobile, only moving slightly when food adjacent to their body was exhausted. Although stage J1 can be reached without feeding, further development of juveniles was not possible without feeding them the sponge *Dysidea* sp.

##### Stage J3 -36 days post hatching: mantle pigmentation appears

At the start of juvenile stage three (J3), the mantle became pigmented (Fig. 4B, C). Yellow pigmentation became visible around the margin of the mantle and at the dorsal midline, while blue pigmentation appeared within inner mantle region. At the end of stage J3, intestinal peristalsis was visible through the mantle, showing that the juveniles were able to feed (Supplementary Video 5). However, excretion of intestinal contents from the juvenile anus was no longer observed. As a result, the body’s posterior thickened, leading to near immobile individuals.

#### Mid juvenile phase: Development of major adult organs

##### Stage J4—42 days post hatching: formation of adult anus and anal papilla at the posterodorsal side

**Figure 6:**
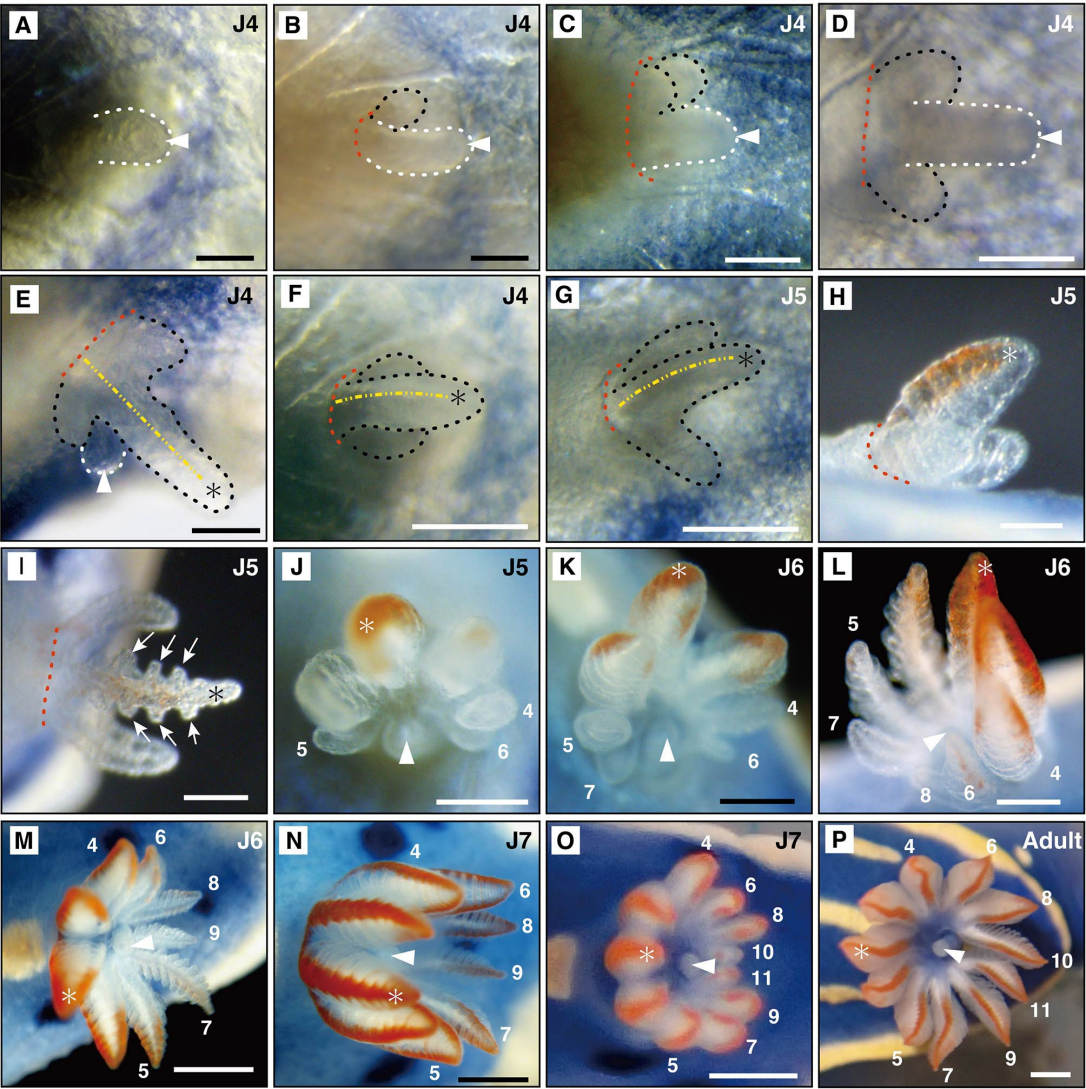
Formation of dorsal anal papilla and gills at stages in *H. festiva*. (**A**) Anal papilla (arrowhead and white dotted line) at the posterodorsal part of the mantle. (**B**) Gill cavity (red dotted line indicates edge of gill cavity) present around the anal papilla. The first gill plume (black dotted line) develops to the right of the anal papilla. (**C**) An additional plume develops to the right of the anal papilla. (**D**) A plume is present on either side of the anal papilla. (**E**) Gill with three plumes present anterior to the anal papilla. The yellow dotted line shows the midline of the gill. (**F**) The gills are slightly bent from the base posteriorly and cover the anal papilla. (**G**) The three gill plumes form an arc surrounding the anal papilla. (**H**) Vermilion pigmentation occurs at the longest plume (*). (**I**) Pinnules (white arrows) begin to develop on the plumes. (**J**) New plumes are added alternately to the right and left ends of the arc. Numbers indicate the order of formation of the new plumes. (**K**) Seven plumes, and pigmentation spreads. (**l**) Eight plumes with pigmentation seen in six plumes. (**M**) Nine plumes with the plumes at both ends of the arc in contact forming a circle. (**N**) All nine plumes have pinnules and pigmentation and are uniform in length. (**O**) 10th and 11th plumes appear but are still short. (**P**) The morphology and coloration of the 11 plumes are the same. (**A**–**H**, **J**, **P**) dorsal view, anterior to the left. (**L**): left lateral view, anterior to the left. (**K**–**O**) posterior view. Scale bars: (**A**, **B**) (50 μm), (**C**–**I**) (100 μm), (**J**, **L**) (200 μm), (**K**) (250 μm), (**M**–**O**) (500 μm), P (1 mm). * indicates the longest gill plume at the three plume stage.

Juvenile stage four (J4) was characterized by the adult anus opening at the tip of the dorsal anal papilla (Fig. 6A, Supplementary Video 6). Excretion from the ventral juvenile anus was not observed, but the juvenile anal gland was still present posteroventrally (Fig. 3D, E). A few days after the formation of the anal papilla, a gill cavity formed around it (Fig. 6B). The papilla was observed to retract into this cavity (Supplementary Video 7). The first gill plume developed to the right of the anal papilla (Fig. 6B). An additional plume also developed to the right of the anal papilla (Fig. 6C), with the following plumes forming bilaterally (Fig. 6D). The anal papilla and gill plumes joined at their respective bases. Three gill plumes then formed anterior to the anal papilla (Fig. 6E). The longest gill plume was folded along the body midline toward the posterior, with plumes slightly bent from the base posteriorly, covering the anal papilla (Fig. 6F). Juveniles were mobile compared to previous juvenile stages, yet mostly stayed situated on *Dysidea* sp. sponges.

##### Stage J5—53 days post hatching: MDFs appear at anterior mantle edge

Juvenile stage five (J5) began when MDFs formed on the lateral sides at the anterior part of the mantle (Fig. 5C). MDFs at the mantle posterior enlarged (Fig. 5D). Gill plumes began to arrange in an arc formation surrounding the anal papilla (Fig. 6G), and vermillion pigmentation and pinnules appeared on the central gill plume (Fig. 6H, I). Subsequently, two new plumes formed on both sides adjacent to each existing plumes (Fig. 6J). Blue pigmentation of the mantle spread in a gradation towards the dorsal midline, and blue spots appeared slightly within the yellow rim of the mantle (Fig. 4D, E). Rhinophores elongated (Fig. 2D) and formed ridges on its surface (Fig. 2E). In the late J5 stage individuals, these ridges were more visible (Fig. 2F). While the arranged spicules observed in stage J1 were still observable (Supplementary Fig. S4C), they were harder to discern in the late J5 stage (Supplementary Fig. S4D). Juveniles occasionally left the sponge and actively crawled around the petri dish.

#### Late juvenile phase: Body size increases, organs develop further, and yellow spots appear. Juveniles are able to lift the anterior part of their bodies.

##### Stage J6—79 days post hatching: rhinophores become spindle shaped

Spindle shaped rhinophores marked the start of juvenile stage six (J6) (Fig. 2G, H). Rhinophores reached peak thickness at about one-third from their base and possessed lamellae, thin plate-like structures, derived from surface ridges. The morphology of the rhinophores was similar to that of adults, with lamellae further increasing with growth. Gill plumes increased in succession to nine and arranged in a circular formation (Fig. 6K–M). Each plume acquired pinnules and vermilion pigmentation, thus acquiring the same morphology as adult gill plumes. Spicules were no longer visible from the dorsal side. Blue pigmentation spread over the entire mantle and foot (Fig. 4F), with additional blue spots forming near the dorsal midline (Fig. 4G). Juveniles were active and sometimes lifted the anterior part of their body from substratum.

##### Stage J7 -94 days post hatching: appearance of yellow spots

The appearance of dorsal yellow spots indicated that the juveniles had reached the final juvenile stage (J7) (Fig. 4H). In the early phase of this stage, juveniles had nine pigmented gill plumes of the same length (Fig. 6N). Subsequently, the tenth and eleventh gill plumes appeared but were small compared to the first nine plumes (Fig. 6O).

#### Adult phase: Mating behavior and spawning can be observed.

##### Stage Adult -164 days post hatching: gills have 11 plumes of the same length

When the length of the tenth and eleventh gill plumes were the same as the previous nine (Fig. 6P), individuals were deemed to have reached adulthood. During this stage, the number of gill plumes further increased. This was consistent with a previous report of adult *H. festiva*, which can possess eleven to thirteen gill plumes^41^. Adult individuals actively approached one another for mating and subsequent egg laying. Individuals that hatched in May 2020 and were reared in the laboratory mated and laid eggs 462 and 492 days after hatching, respectively. Individuals that hatched in May 2021 and reared in the laboratory mated 164 days and laid eggs 175 and 183 days after hatching.

## Discussion

This study is the first report of chromodorid development, from embryos to adults in the laboratory. The post-settlement growth process of *H. festiva* was divided into nine stages based on external morphological changes (Table 1). This enables researchers to stage juveniles collected from the field without the need for dissections or manipulative experimentation. During *H. festiva*’s metamorphosis phase M1, post-settlement morphological changes occurred in the following order: degeneration of the velum, casting of the operculum, and casting of the shell (Fig. 1, Supplementary Video 2). The order of these events is identical to other sea slug species that lose their shells during metamorphosis^25,27,28,36^. In stage M2, the posterior end of the mantle is initially divided into two lobes. This was consistent with the previous report of metamorphosis from the onchidoridoidean *Adalaria proxima*^24^. The same has also been reported for the sea slug *Pleurobranchaea japonica* within Pleurobranchida, the sister group of Nudibranchia^44^. Previous reports on stage M2 from nudibranchs including the Doridina species *H. infucata*^36^, *Rostanga pulchra*^28^, and *Onchidoris bilamellata*^22,33^, and the Cladobranchia species *Phestilla sibogae*^25^ and *Melibe leonina*^27^ lack descriptions of the posterior mantle tip morphology. More detailed observations on the metamorphosis of these and other species may reveal that mantle formation via a two-lobed posterior stage is a common occurrence across other nudibranchs species.

Nudibranch anuses are usually present on the body’s dorsal or posterior side after metamorphosis depending on the species^24,25,27,45,46^. Although the position of this anus may gradually move during growth, it is regarded to be maintained and used continuously into adulthood^8,46,47^. However, the *H. festiva* juveniles in this study showed the juvenile anus was present on the ventral side (stages J1 to J3; Fig. 3), while the adult anus opened at the tip of the dorsal anal papilla (stage J4; Fig. 6A). Similar anus positions have also been reported in the dorid sea slug *Cadlina laevis* of the family Cadlinidae^26,48,49^, but the detailed process in which the anus changes its position was not observed. It has been speculated that the anus gradually moves during *C. laevis* juvenile growth^48^. However, in *H. festiva* J4 juveniles already possessing a functional dorsal adult anus, the cells of the anal gland that surrounded the juvenile anus remained at the ventral mantle (Fig. 3D, E). This implies that the anus and the tissues around the juvenile anus did not move dorsally. Based on this observation, we hypothesize that the adult anus is formed independently from the juvenile anus during *H. festiva* ontogeny. The same digestive organs, including the stomach and digestive glands, are used in both juvenile and adult stages. As such, we assume that the distal end of the intestines detaches from the ventral juvenile anus, migrate, and connect with the dorsal adult anus during the juvenile stages. At the late J3 stage, no excretion was observed from either anus for several days, suggesting that the migration of the intestine tip could have occurred during this time.

**Figure 7:**
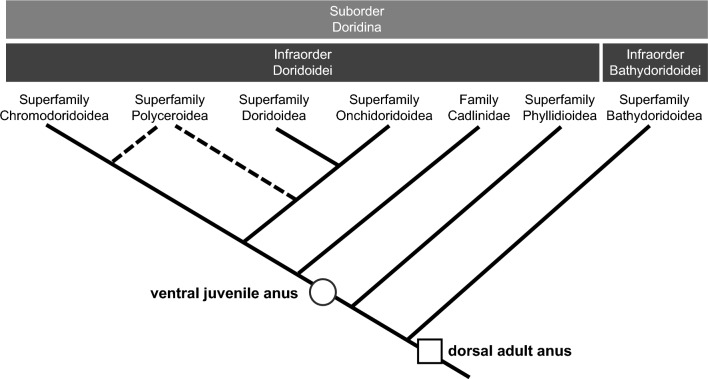
Hypothesis on the juvenile anus in the suborder Doridina. The topology is based on previous research^51,52^. It is widely accepted that a dorsal adult anus was present in the last common ancestor of the suborder Doridina (square). We suggest the possibility that a ventral juvenile anus followed by a dorsal adult anus was present in the common ancestor of the infraorder Doridoidei excluding the superfamily Phyllidioidea (circle).

There have been reports of juveniles corresponding to stages J1 to J3 from several other dorid species^17,22,28,31,33–35^. The anus in the adults of these species are positioned dorsally. However, the location of the anus in juveniles was not clarified in these descriptions and diagrams^17,22,28,31,33–35^. The only exception is from the Onchidoridoidea *Corambe steinbergae*, whose juveniles and adults both possess a ventral anus^50^. As mentioned earlier, the anus is positioned ventrally in juveniles of *C. laevis*^26,48,49^ and *H. festiva* (Fig. 3). In other words, in the suborder Doridina estimated to contain more than 1,500 species^10^, the presence of a juvenile anus has only been examined for three species, *H. festiva, Cadlina laevis,* and *Corambe steinbergae*, where all three possessed a ventral juvenile anus. We cannot rule out the possibility that a ventral juvenile anus was acquired independently in these three species. However, when the currently accepted phylogenetic hypotheses of dorids are considered^51,52^, it is also possible that a ventral juvenile anus was present in the last common ancestor of these three species, namely the last common ancestor of the infraorder Doridoidei excluding the superfamily Phyllidioidea (Fig. 7).

A few species of the family Corambidae in the superfamily Onchidoridoidea have an anus on the ventral side even in adults^53^. Given that nearly all species in the infraorder Doridoidei have adult dorsal anus and considering the phylogenetic relationships among these groups (Fig. 7), we assume that the last common ancestor of the infraorder Doridoidei had a dorsal adult anus, whereas the few species in the superfamily Onchidoridoidea acquired a ventral adult anus secondarily. If the last common ancestor of the infraorder Doridoidei excluding the superfamily Phyllidioidea had a ventral anus during juvenile stage as suggested above, it raises the possibility that the aforementioned Onchidoridoidea species with a ventral adult anus acquired the trait due to neoteny.

MDFs are first formed at the posterior mantle edge at the J2 stage (Fig. 5A, B), shortly after juveniles began feeding. This is consistent with the functional property of MDFs; to store food-derived chemicals and secondary metabolites^16^. The arrangement of MDFs is a taxonomic trait in the chromodorids, but some studies caution against using only this trait for classification^54^. In this study, we showed that the placement of MDFs is influenced by ontogenetic stages, further implying the risk of using only MDFs arrangement for species identification.

The gill plumes of adult dorids are typically arranged in semi-circles or circles depending on the species^8^, and the morphology of the gills is a taxonomic trait used for distinguishing between closely related species^55^. The gill plumes of *H. festiva* changed from an arc to a circular arrangement during its formation (Fig. 6), with the same phenomena also reported for the dorid *Adalaria proxima*, belonging to the superfamily Onchidoridoidea^24^. These studies suggest that the morphology of the gills can change during ontogeny, and similar to MDFs, should not be used exclusively for identification. Taken together, as gill morphology and arrangement of MDFs can change during sea slug growth, it is essential to use multiple external characteristics when identifying chromodorid species.

Juvenile and adult *H. festiva* have been reported to show different body color patterns^41^, yet these observations have only been on field collected individuals. Here we show that blue and yellow pigments appeared during the juvenile stage J3, with lines and spots forming sporadically across these stages throughout development (Fig. 4). Juvenile *H. festiva* reported in Baba (1995)^41^ had blue pigmentation on the foot and mantle, a single yellow line on the dorsal midline, and no yellow spots, thus corresponding to stage J6 in this study. This demonstrates the utility of our categorized stages as a comparison to previous sample specimens, as external characteristics, including body coloration, can be easily delimitated throughout this chromodorid’s development.

## Conclusion

Developmental research on chromodorids has stalled for the last few decades as they are difficult to incubate and rear. Here, we report finely detailed methods for culturing *H. festiva* from eggs to adults in a laboratory, and describe its development post-settlement into nine stages using only external observations. With these established methods and their abundance throughout mainland Japan, we propose *H. festiva* as a model organism for future studies on chromodorid development. The methods, staging, and data presented in this study could be implemented to other species and will form the basis for further developmental research on chromodorids. We hope that this research will enable the application of various research techniques such as genome editing and establishment of transgenic lines on chromodorids, thus contributing to the elucidation of the molecular basis of organ and body color formation of chromodorids.

## Materials & methods

### Collecting animals

The nudibranch *Hypselodoris festiva* (A. Adams, 1861)^40^ and its sponge prey *Dysidea* sp. (Supplementary Fig. S5A) were collected by hand from tide pools in the intertidal zone and by SCUBA diving at a depth of 2 to 15 m along rocky wall overhangs at Nabeta Bay (Shimoda, Shizuoka, Japan). Animals were identified based on external characteristics. Five individuals of *H. festiva* were collected during each of the breeding seasons in 2020 and 2021 for use in this study. Collection was conducted in compliance with local laws with scientific collection permits issued by Shizuoka Prefecture, Japan.

### Rearing

Antibiotic–Antimycotic Solution (Penicillin–Streptomycin-Amphotericin B Suspension; × 100; 10,000 units/ml Penicillin G, 10,000μg/ml Streptomycin sulfate, 25μg/ml Amphotericin B; FUJIFILM Wako Pure Chemical Corporation, Japan) was diluted 1000 times with natural sea water filtered using vacuum filtration (Filtermax, TPP, Switzerland). This antibiotic-added filtered sea water (AFSW) was used for incubating egg masses, larvae, juveniles, and adults. All rearing was carried out at 22°C unless otherwise noted.

#### Preparing sponges

As *Hypselodoris* has been reported to feed on *Dysidea* sponges^39^, four different *Dysidea* groups found at the *H. festiva* collection sites were tested as food for *H. festiva*. While we were unable to classify the four *Dysidea* groups to the species level, they all showed distinct external characteristics for which we assumed indicated they different species. When placed into containers together with *H. festiva*, individuals showed preference for one of the four *Dysidea* groups. When whole specimens of this *Dysidea* sp. were fed to *H. festiva*, polychaetes inhabiting the sponge often attacked the nudibranch during feeding. This issue was solved by chopping and washing the sponge prior to feeding (Supplementary Fig. S5B). Sponges about 10 cm in diameter were maintained in an overflow natural sea water tank system before use. They were shredded into small pieces around 5 mm in diameter using a hand-cranked mincer, washed with AFSW four to five times daily for three days, and maintained in a sieve bowl filled with AFSW (Supplementary Fig. S5B).

#### Obtaining egg masses

Adults of *H. festiva* were maintained in 5 L glass tanks filled with AFSW and constant aeration. Two to five individuals were maintained in the same tank to induce mating. The sponge *Dysidea* sp. was fed directly after changing tank water, which was changed every three days.

#### Egg masses

After spawning, egg masses were detached from the tank walls using a pipette tip or razor blade and transferred to petri dishes. Debris and organisms on the mucus around the masses were washed away. As we observed the underdevelopment of larvae within the inner layer of *H. festiva*’s egg masses (possibly due to hypoxia), the masses were chopped into small pieces about 2–5 mm wide using dissection needle and pipette tip before incubation (Supplementary Fig. S5C). These pieces were placed into plastic dishes (diameter 90 mm) filled with AFSW.

#### Larval culture

After hatching, larvae were collected with micropipettes and transferred to 5 L beakers. Around 2,000–2,500 larvae were bred in each beaker with 4 L of AFSW (Supplementary Video 1). The water was gently stirred by a stirrer powered by a low-speed synchronous motor constantly (Supplementary Fig. S5D). Because many larvae adhered to the water surface, a second propeller was attached at the water surface to the beakers’ stirrers, which freed the trapped larvae by constantly creating small waves. When the synchronous stirring motor was set at 30 rotations per minute (rpm), the speed commonly used for rearing marine invertebrate larvae^56,57^, the larvae were unable to feed. We suspected that the water movement at 30 rpm is too strong for the larvae, causing them to close their operculum. The larvae were able to feed when the motor was slowed to 5 rpm. The larvae were fed *Chaetoceros* sp*.* every one to three days. 3 L of ASFW were changed every 2 to 3 days by using 55 μm or 100 μm nylon mesh filters, depending on the larval size.

#### Metamorphosis

Larvae that had developed a foot were collected from the beakers and were transferred to glass dishes (90 mm diameter) using 100 μm nylon mesh filters and micropipettes. Two methods of collection were used depending on the quantity of larvae. When large numbers of larvae were present, the entire contents of the beakers were passed through a 100 μm nylon mesh filter, and larvae remaining in the filter was collected by a glass volumetric pipette. When there were only a small number of larvae, individuals were collected directly from the beakers with either a glass volumetric pipette or a P1000 micropipette outfitted with a low adsorption tip (platinum chip, BMBio, Japan). To assist with larvae collection, beakers and containers were illuminated with a strong light source (e.g. diving light) to force larvae to concentrate near the water surface.

#### Juvenile stage

A small piece of the sponge *Dysidea* sp. was placed in plastic dishes (diameter 50 mm) containing AFSW, and the sponge adhered to the bottom and sides of the dishes after a few days (Supplementary Fig. S5E). Individuals that settled and metamorphosed were transferred to these dishes and kept individually. The water was changed every other week. Juveniles with a body length of 5 mm or longer were transferred from the dishes to 300 ml plastic bottles with aeration and kept individually. Water was changed every three days and pieces of sponge about half to one-third of the juvenile body length were fed just after the water change. When body length reached about 1.2 cm, multiple individuals were bred together in 5 L aerated glass tanks. Individuals were fed *Dysidea* sp. once every week, and the water was changed every two days after feeding.

### Observation of external morphology

Larvae and juveniles up to 7 mm were observed using a stereomicroscope (Leica M205 C, Germany) and recorded with a digital microscope camera (AdvanCam-18HRII, Japan). Recording of juveniles larger than 7 mm was performed using digital cameras (OLYMPUS Tough TG5 and TG6, Japan) in a hand-made 30 × 30 × 15 cm light box. A hole the same size as the lens of a digital camera opened at the top of the box, and two white LED lights were installed inside the box, assuring that the light source and intensity was constant during photography of this study. Analyses of the photographs were performed using the software attached to the camera (AdvanView 3.7, Japan) and Adobe Photoshop CC.

### Larval growth

Larval shell lengths (µm) were measured following Pires (2024)^58^. Supplementary Fig. S3B was produced using R software v4.0.0^59^. A linear model (lm) was used to describe the relationship between larval shell length (µm) and days post hatching. A best-fit second-order polynomial regression line was used following Trowbridge (2000)^60^ to demonstrate the plateauing of early-stage growth patterns.

### Supplementary Information

Supplementary Information 1.

Supplementary Video 1.

Supplementary Video 2.

Supplementary Video 3.

Supplementary Video 4.

Supplementary Video 5.

Supplementary Video 6.

Supplementary Video 7.

## Data Availability

The datasets used and/or analyzed during the current study are available from the corresponding authors on reasonable request.
