# Supplementary material for: Staging of post-settlement growth in the nudibranch Hypselodoris festiva
Source: Sci Rep. 2024 Jul 21;14:16157. doi: 10.1038/s41598-024-66322-4 (PMC11271472; doi:10.1038/s41598-024-66322-4)
Supplement: Supplementary file 1 — Supplementary Information 1. [file 41598_2024_66322_MOESM1_ESM.pdf]

Staging of post-settlement growth in the nudibranch *Hypselodoris festiva*

Makiko Hayashi[1*], Hiroaki Nakano[1*]

1. Shimoda Marine Research Center, University of Tsukuba, 5-10-1 Shimoda, Shizuoka, Japan

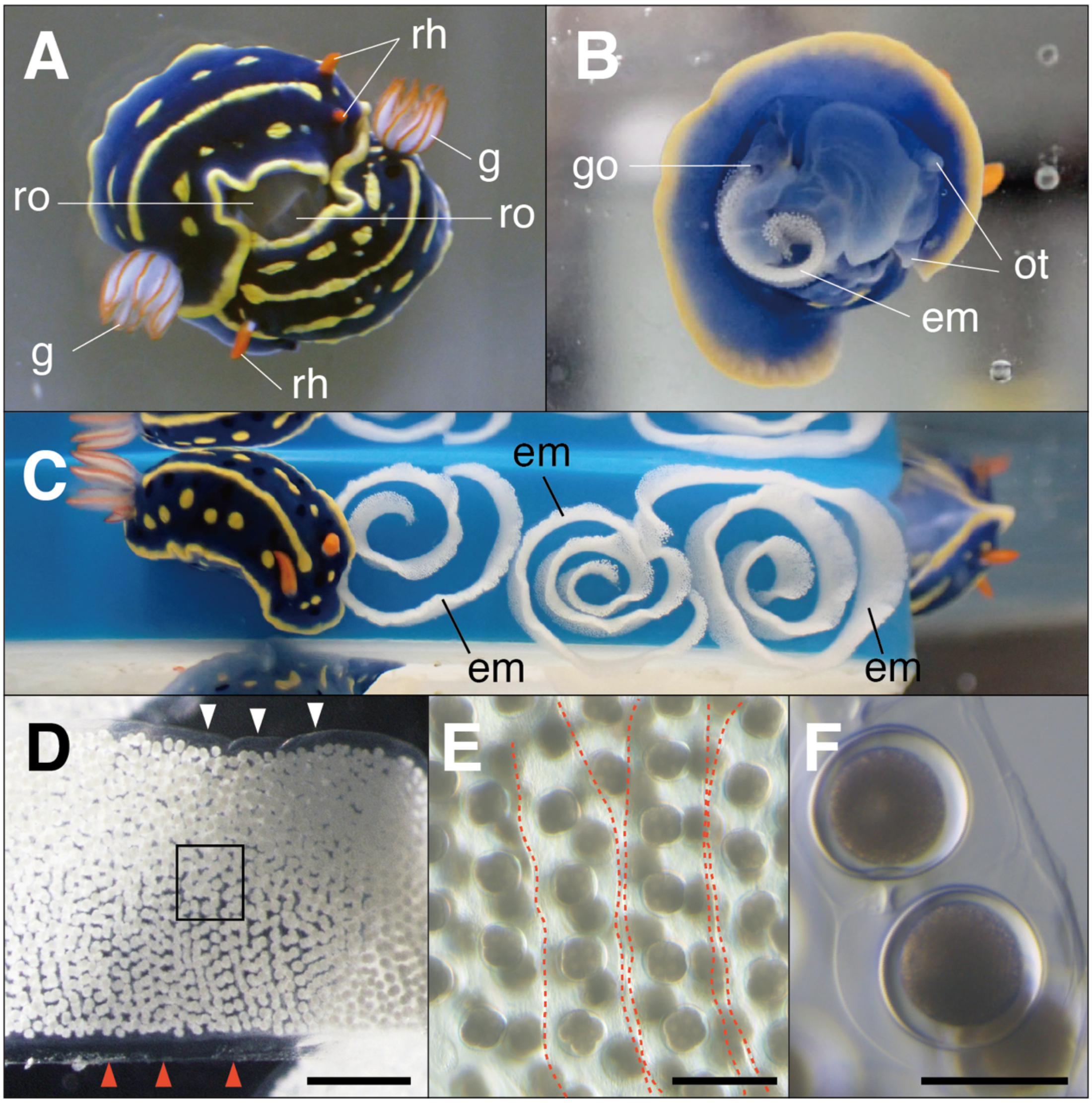

Supplementary Figure S1- Mating and spawning of *H. festiva*

A: Adult individuals during mating. B: Ventral view of an individual spawning. C: Three egg masses laid in an aquarium. D: Close up of an egg mass. Red arrowheads: attached edge of the egg mass, white arrowheads: free edge. E: Close up of the area shown with a rectangle in D. Eggs form rows vertical to the edges. Red dotted lines outline some of the rows. F: Eggs in an egg mass. Abbreviations: em (egg mass), g (gill), go (gonopore), ot (oral tentacle), rh (rhinophore), ro (reproductive organ). Scale bars: D (1 mm), E (200 μm), F (100 μm).

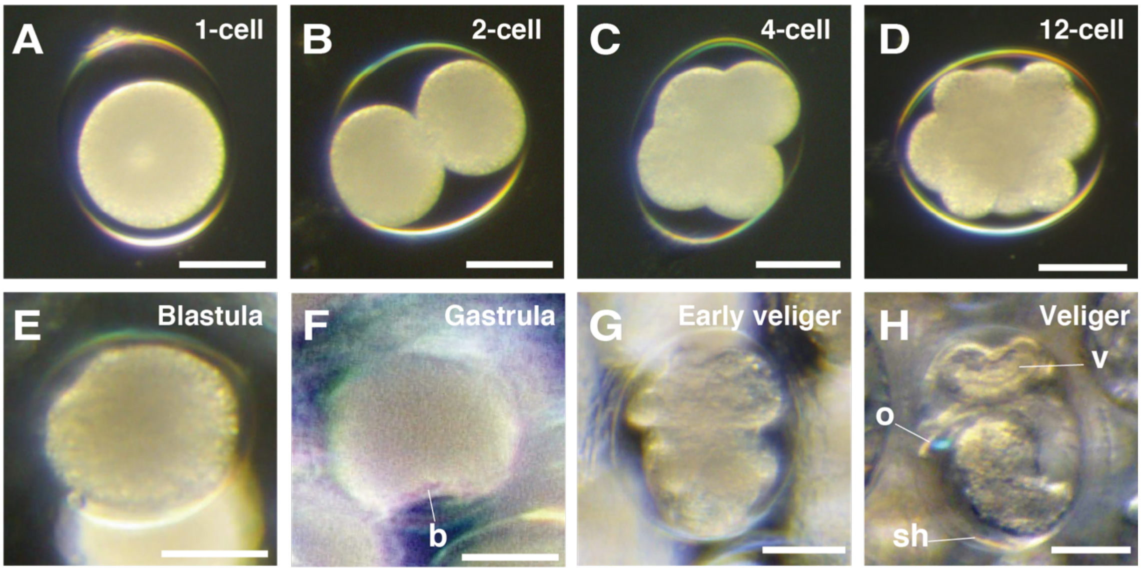

Supplementary Figure S2- Embryonic development of *H. festiva*

Abbreviations: b (blasotopore), o (operculum), sh (shell), v (velum). Scale bars: (50 µm).

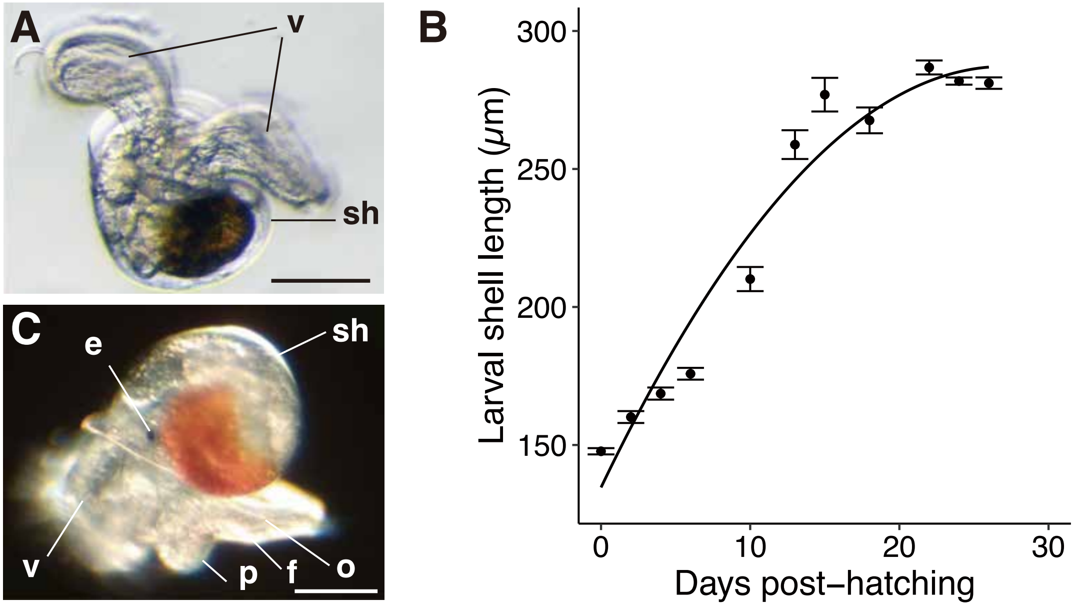

Supplementary Figure S3– Larval development of *H. festiva*

A: Veliger larva 13 days post-hatching, ventral view, anterior to top. B: Larval growth at 22°C. Dots indicate the average shell length, and error bars indicate standard error. C: Pediveliger larva 34 days post-hatching, left lateral view, anterior to the left. Abbreviations: e (eye spot), f (foot), o (operculum), p (propodium), sh (shell), v (velum). Scale bars: (100 µm).

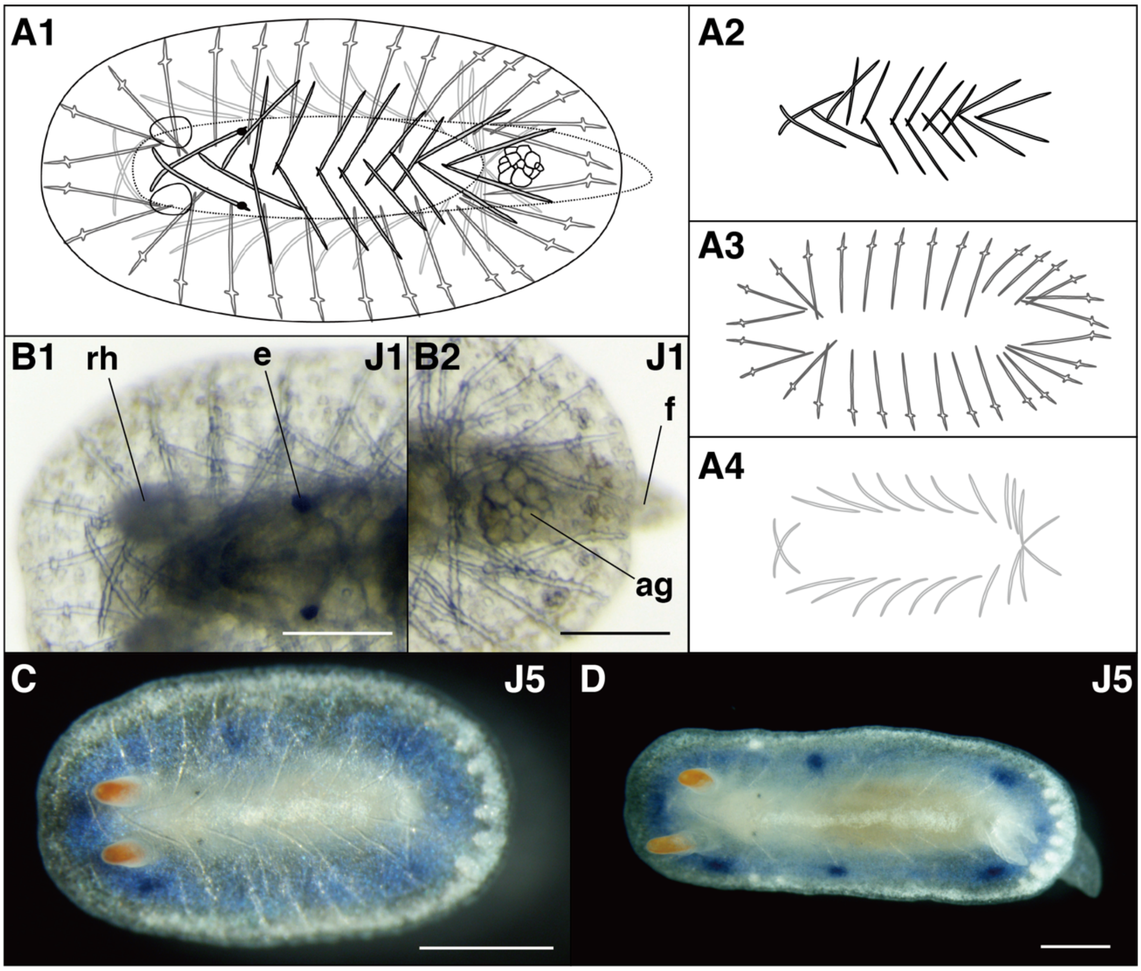

Supplementary Figure S4- Juvenile spicules of *H. festiva*

A1-A4: Diagram of the dorsal view of major spicules in the J1 juvenile. A1: Overview. A2-4: Diagrams showing spicules in the most dorsal (A2), middle (A3), and ventral (A4) parts of the mantle. B1: Anterior, and B2: posterior part of a J1 juvenile. C: A juvenile at stage J5. D: Same individual as C, 20 days later. A-D: dorsal view, anterior to the right. Abbreviations: ag (anal gland), e (eye spot), f (foot), rh (rhinophore). Scale bars: B1 and B2 (100 µm), C and D (500 µm).

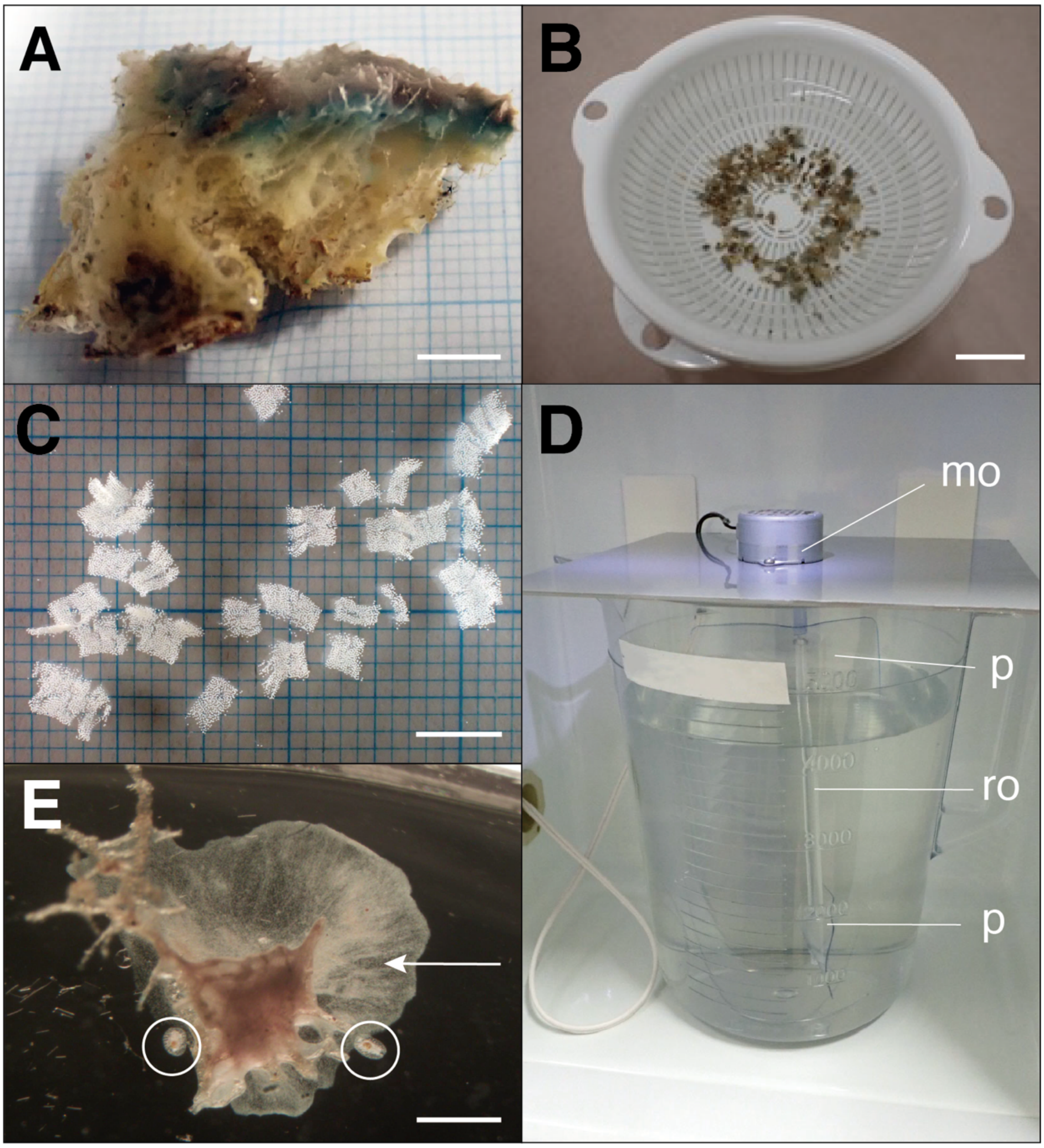

Supplementary Figure S5- Rearing of *H. festiva*

A: Prey sponge *Dysidea* sp. B: Chopped sponge maintained in a sieve bowl before use. C: Chopped egg mass. D: Larval culture system with an additional propeller at the water surface. mo (synchronous motor), p (propeller), ro (rotor) E: Juveniles seven days after settlement. Two juveniles are present in this dish. Circles: juveniles; arrow: the sponge *Dysidea* sp. adhered to the bottom of the dish. Scale bars: A and C (5 mm), B (3 cm), E (1 mm).

Supplementary Video Legends:

Supplementary Video 1 - Swimming *H. festiva* larvae 15 days after hatching

Supplementary Video 2 - *H. festiva* metamorphosis during stage M1

Supplementary Video 3 – *H. festiva* stage J1 juvenile with paired retractable rhinophores

Supplementary Video 4 - Excretion from the ventral juvenile anus of a *H. festiva* stage J2 juvenile

Supplementary Video 5 - Peristaltic motion of the intestine in a *H. festiva* juvenile during the last few days of stage J3

Supplementary Video 6 - Excretion from the adult anus at the tip of the anal papilla in a *H. festiva* stage J4 juvenile

Supplementary Video 7 - Retraction of the papilla and gill plume into the gill cavity in a *H. festiva* stage J4 juvenile
